# Pain Management in Paediatric Critical Care: A Cross‐Sectional Study

**DOI:** 10.1111/nicc.70327

**Published:** 2026-01-20

**Authors:** Elyse Laures, Cynthia LaFond, Ann Marie McCarthy

**Affiliations:** ^1^ University of Iowa Health Care Iowa City Iowa USA; ^2^ Ascension Health St. Louis Missouri USA; ^3^ University of Iowa College of Nursing Iowa City Iowa USA

**Keywords:** barriers and facilitators, paediatric critical care, pain assessment, pain management

## Abstract

**Background:**

Up to 24% of children admitted to the paediatric intensive care unit (PICU) experience moderate to severe pain. Nurses have the ethical responsibility to manage pain, which is often challenging due to a wide range of age, developmental ability, medical diseases and complex medical equipment. Limited research has been done, specifically in the PICU, related to pain management practices and barriers and facilitators.

**Aim:**

The purpose of this study is to describe PICU nurses' current pain assessment and analgesic premedication practices, in addition to facilitators and barriers to pain management.

**Study Design:**

This study is a secondary analysis of a previous cross‐sectional study. PICU nurses were asked to complete a survey regarding their pain assessment practices, premedication practices, perceptions of patient behaviours indicative of pain and barriers and facilitators.

**Results:**

A total of 106 nurses responded. It was found that nurses more routinely use behavioural assessment scales compared to self‐report and do not regularly attempt to obtain a self‐report for those who are mechanically ventilated. The decision to premedicate prior to a painful procedure was variable. Nurses found only four patient behaviours as most commonly indicating pain. Lastly, facilitators were perceived more commonly present than barriers, with nurses valuing having a designated charting system and standardised assessment tools.

**Conclusion:**

Overall, there is a need to leverage facilitators and minimise barriers to successfully implement evidence‐based pain management practices. Future research in the creation of clinician support tools, following the principles of implementation science, is needed.

**Relevance to Clinical Practice:**

Understanding current pain management practices better enables frontline nurses, educators and leaders to identify, implement and sustain evidence‐based pain management improvements.

## Impact Statements

- What is known about the topic?
  ○ Children seen in the paediatric intensive care unit (PICU) are known to experience pain and nurses are critical to assess and manage pain in a vulnerable population.
  ○ There is a wide variation in how nurses assess and manage pain across all children seen in the PICU and the degree to which evidence‐based practices are used.
- What this paper adds?
  ○ This study adds further variation in practices not previously reported, such as the use of pain medication prior to procedures and asking for a self‐report assessment for a child who is mechanically ventilated.
  ○ Key facilitators such as the use of protocols or guidelines and clear charting areas within the electronic health record were identified and should be used as leverage to better implement evidence‐based practices at the bedside.

## 1 Introduction

### 1.1 Background

Critically ill children continue to experience pain and its consequences today. In a recent study, 45% of children in the paediatric intensive care unit (PICU) experienced pain, and 24% had moderate to severe pain during a 24‐h timeframe [1]. Additionally, it has been reported that children in the PICU experience more severe pain compared to children on other paediatric units and uncontrolled pain is the number one preventable adverse event reported in the PICU [2, 3]. In the short term, unrelieved pain can cause acute physiological changes, altered sleep patterns and weakened immune functions that can result in increased length of hospital stay and increased hospital costs [4, 5, 6]. In the long term, inadequate pain management while hospitalised in the PICU is a risk factor for developing post‐intensive care syndrome‐paediatrics (PICS‐p) [7, 8]. PICS‐p is a complex syndrome that includes the physical, emotional, cognitive and psychosocial impairments experienced following PICU discharge [8, 9, 10]. With the rise of medical complexity seen in children in the PICU [11], it is increasingly important to manage pain appropriately while in the PICU.

The American Nurses Association states that ‘Nurses have the ethical responsibility to relieve pain and the suffering it causes’ ([12, p. 1]). Moreover, they are the frontline for management and monitoring of pain for children in the PICU [13]. However, managing pain in the PICU is complex due to the varying levels of communicative ability, age, developmental level, use of medical interventions/equipment and severity of critical illness [14, 15, 16]. In order to effectively assess pain, nurses must be familiar with a multitude of pain assessment methods and scales [15], leading to increased cognitive load [17]. There are also many organisational factors that contribute to the nurse's ability to manage pain effectively, such as training, interdisciplinary collaboration and workload [18, 19].

Further impacting the nurse's ability to appropriately manage pain is the complex intersection among pain, agitation, iatrogenic withdrawal and delirium. Specifically, nurses must continuously manage both pain and sedation while also trying to minimise the unintended consequences of iatrogenic withdrawal and delirium. Often the signs of each are overlapping and similar [14, 20]. While there are a variety of protocols to manage pain and sedation in critically ill adults and children, their implementation remains inconsistent for a variety of reasons, such as complexity, organisational infrastructure or lack of an effective implementation plan [21, 22, 23]. Implementation strategies need to be developed that fill the gaps in practice [24, 25]. Understanding current practices from end users, including facilitators and barriers encountered, is an essential first step. While there have been various reports related to pain management practices and facilitators and barriers for neonates [26], critically ill adults [18, 27] and the general paediatric population [28], little is known about practices specific to the PICU. It remains critical to understand what is happening and what is helping to manage pain for this critical patient population.

### 1.2 Aim

The purpose of this secondary analysis is to describe PICU nurses' current pain assessment and analgesic premedication practices. In addition, facilitators and barriers to pain management for the complex children seen in the PICU are described. The rationale for this analysis was to investigate how nurses are managing pain for all children in the PICU to be able to identify areas to improve through implementation of targeted interventions. This will enable the development or adaptation of tools, algorithms and protocols to manage pain for the frontline nurse.

## 2 Study Design and Methods

### 2.1 Setting and Sample

This study is a secondary analysis of data from a cross‐sectional quantitative study that aimed to describe PICU nurses' decision making when managing pain specifically for children who are mechanically ventilated and chemically paralysed [29, 30]. Self‐report data was collected from PICU nurses nationally. Nurses were recruited through multiple list serves, including the National Pediatric Nurse Scientist Collaborative [31], the Society of Pediatric Nurses and a PICU nursing science group. Eligibility criteria included: English speaking with at least 6 months of experience as a staff nurse in a PICU [30]. Due to our recruitment method, the number of respondents the surveys were sent to (to obtain a denominator) is not known.

The primary study reported only pain assessment decision making for children who are mechanically ventilated and chemically paralysed [30] via four written vignettes at the start of the survey. Following the vignettes, additional questions were asked pertaining to general pain management for all children seen in the PICU. Therefore, this secondary analysis includes data from the original study describing the PICU nurses and their general pain assessment and analgesic premedication practices in caring for all PICU patients and also their barriers and facilitators when managing pain.

### 2.2 Data Collection Tools and Methods

In the original study, data collection occurred from August 2020 to January 2021. Upon ethics approval, eligible PICU nurses received the study information via their emails with a link to complete the survey. REDCap (Research Electronic Data Capture), an electronic data capture tool, hosted at the University of Iowa [32], was used to collect and store data [30].

#### 2.2.1 Survey

Included in this study are the survey results for: nurse demographics, pain management practices and facilitators and barriers to pain management. Each section of the survey had clear break points and introductory content to ensure clarity of each of the questions (Data S1).

Demographics included the nurses' age, ethnicity/race, gender, experience, level of education and work shift as well as their PICU type, size and region.

##### 2.2.1.1 Pain Management Practices and Barriers and Facilitators

Pain management practices and facilitators and barriers were collected by adapting, with permission, the Pain Assessment and Management of the Critically Ill Adult Survey [27] to fit the critically ill paediatric population. The revised survey was tested for clarity, content validity and comprehensiveness by 10 content experts [30]. The survey included 10 items related to pain assessment practices ranging from multiple choice to select all that apply, 7 items on analgesic premedication practices on a 5‐point Likert scale, 22 items related to beliefs related to behaviours indicative of pain or agitation, neither or both and 11 facilitator items and 12 barrier items on a 5‐point Likert scale.

### 2.3 Data Analysis

Following data cleaning, descriptive statistics were conducted, including means and frequencies using Microsoft Excel.

### 2.4 Ethical and Institutional Approvals

The University of Iowa Review Board (IRB) approved this study (IRB202006492) prior to its conduction on 6 November 2020. PICU nurses provided their consent by clicking on and completing the survey, which followed the study information sheet.

## 3 Results

For this survey, a total of 106 PICU nurses responded and 95 completed the survey. The decrease in the number who completed the survey can be attributed to survey fatigue as the item response rate dropped as the survey items progressed with demographics being at the end of the survey [30]. As a result, a minimum of 95 respondents are reported for each item with up to 106 respondents on certain items. All responses were included in the study.

### 3.1 Demographics

Of the 95 PICU nurses who completed the entire survey, the ages ranged from 22 to 65 with a mean of 35 (SD = 9.3) years (Table 1). Nurses were more commonly white (*n* = 81, 86.2%), female (*n* = 91, 4.2%), held a bachelor's degree (*n* = 72, 76%), had 2 to 5 years' experience (*n* = 39, 41%) and worked day shift (*n* = 37, 38.9%). The PICUs represented here included mostly general units (*n* = 60, 63.2%), that were 30 to 40 beds in size (*n* = 30, 31.6%) and from the Midwest (*N* = 39, 41.5%) [30].

**TABLE 1:** Demographics.

Variables	Median (Range) or *N* (%)
Age (*N* = 95)	30 (22–65)
Race/ethnicity (*N* = 94)	
White	81 (86.2)
Hispanic/Latinx	5 (5.3)
Black	2 (2.1)
Native American	0
Asian	8 (8.5)
I wish not to specify	2 (2.1)
Gender (*N* = 95)	
Male	4 (4.2)
Female	91 (95.8)
PICU type (*N* = 95)	
General	60 (63.2)
Cardiac	2 (2.1)
Mixed	33 (34.7)
PICU size (*N* = 95)	
Less than 10	9 (9.5)
10 to 20	16 (16.8)
20 to 30	22 (23.2)
30 to 40	30 (31.6)
More than 40	18 (18.9)
PICU region (*N* = 94)	
New England	7 (7.4)
Mid‐Atlantic	6 (6.4)
South	14 (14.9)
Midwest	39 (41.5)
Southwest	2 (2.1)
West	26 (27.7)
How long have you worked in the PICU (*N* = 95)	
Less than a year	3 (3.2)
1 to 2 years	15 (15.8)
2 to 5 years	39 (41.1)
5 to 10 years	15 (15.8)
Greater than 10 years	23 (24.2)
Education level (*N* = 95)	
Associates	3 (3.2)
Bachelors	72 (75.8)
Masters	15 (15.8)
Doctorate	5 (5.3)
Work shift (*n* = 95)	
Days	37 (38.9)
Nights	33 (34.7)
Combination	21 (22.1)
PRN	4 (4.2)

### 3.2 Pain Management Practices

#### 3.2.1 Pain Assessment Practices

**FIGURE 1:**
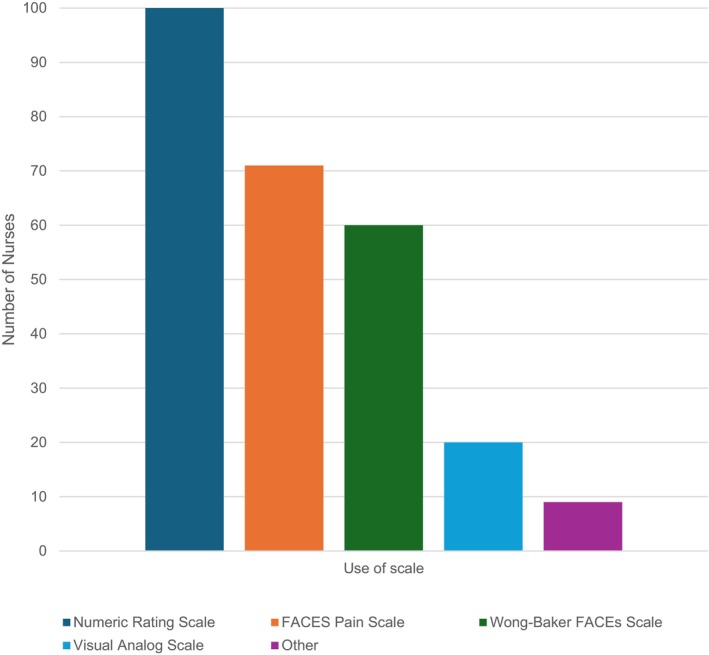
Self‐report scale use (*N* = 103).

Table 2 describes the self‐reported pain assessment practices. All PICU nurses (100%; *N* = 103) reported at some time using an assessment scale for children able to communicate verbally or via other means (holding up fingers, pointing, etc.). Figure 1 reports the most used self‐report pain assessment scales, with most using the scale routinely (82, 78.8%, *N* = 104). When asked if they attempt to obtain a self‐report for a child who is mechanically ventilated, 70 (67.3%) said ‘yes’.

**TABLE 2:** Pain assessment practices.

Children able to communicate (verbally or via other means [holding up fingers, pointing, etc.])		
Item	Yes *N* (%)	No *N* (%)
Do you use a pain assessment scale for patients able to communicate? (*N* = 103)	103 (100)	0
Do you attempt to obtain a self‐report of pain for a child that is mechanically ventilated? (*N* = 104)	70 (63.3)	34 (32.7)

Item	Never *N* (%)	Seldom *N* (%)	Sometimes *N* (%)	Often *N* (%)	Routinely *N* (%)
How frequently do you use a pain assessment scale in routine practice for those able to communicate? (*N* = 104)	0	1 (1)	4 (3.8)	17 (16.3)	82 (78.8)

Children unable to communicate (verbally or via other means [age, sedation, pharmacological paralysis, development delay, etc.])		
Item	Yes N (%)	No N (%)
Do you use a pain assessment scale for patients unable to communicate? (*N* = 104)	100 (96.2)	4 (3.8)

Item	Never *N* (%)	Seldom *N* (%)	Sometimes *N* (%)	Often *N* (%)	Routinely *N* (%)
How frequently do you use a pain assessment scale in routine practice for those unable to communicate? (*N* = 104)	0	1 (1)	0	12 (11.5)	91 (87.5)

**FIGURE 2:**
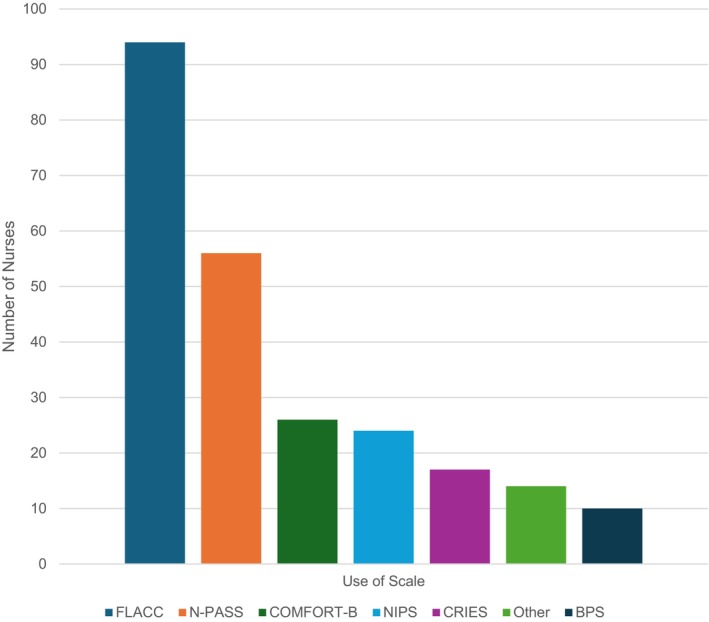
Behavioural scale use (*N* = 100).

**FIGURE 3:**
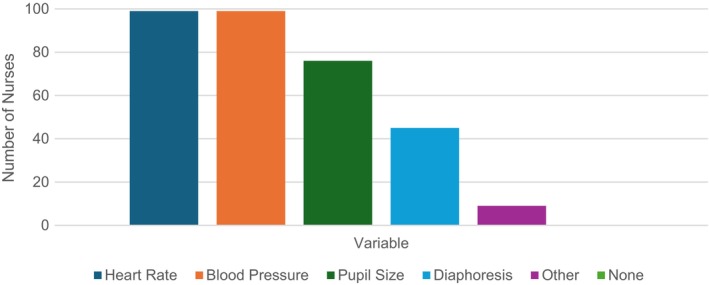
Reported perceived physiological variables indicative of pain (*N* = 99).

Next, most PICU nurses (96.2%; *N* = 104) reported at some time using a pain assessment scale for children unable to communicate verbally or via other means (age, sedation, pharmacological paralysis, developmental delay, etc.), with most using it frequently (91, 87.5%, *N* = 104). Figure 2 shares the most frequently used behavioural assessment scales. Lastly, Figure 3 reports the most commonly selected physiological variables that PICU nurses felt were indicative of pain; other responses included respiratory rate and oxygen saturation.

#### 3.2.2 Premedication Practices

Table 3 reports current practices of PICU nurses when they deliver premedication for pain relief prior to procedures. Invasive line placement (*N* = 94, 88.7%), wound care (*N* = 45, 42.5%) and drain removal (*N* = 41, 38.7%) were the procedures in which nurses most routinely premedicated.

**TABLE 3:** Analgesia premedication practices.

Item (*N* = 106)	Never *N* (%)	Seldom *N* (%)	Sometimes *N* (%)	Often *N* (%)	Routinely *N* (%)
Spontaneous breathing trial	57 (53.8)	42 (39.6)	4 (3.8)	2 (1.9)	1 (0.9)
ET suctioning	13 (12.3)	55 (51.9)	25 (23.6)	12 (11.3)	1 (0.9)
Patient repositioning (side to side)	10 (9.4)	49 (46.2)	32 (30.2%)	13 (12.3)	2 (1.9)
Imaging	3 (2.8)	44 (41.5)	35 (33)	22 (20.8)	2 (1.9)
Drain removal	1 (0.9)	6 (5.7)	12 (11.3)	46 (43.4)	41 (38.7)
Wound care	0	2 (1.9)	10 (9.4)	49 (46.2)	45 (42.5)
Invasive line replacement	0	0	0	12 (11.3)	94 (88.7)

#### 3.2.3 Behaviours Indicative of Pain and Agitation

Lastly, PICU nurse perceptions of behaviours indicative of pain, agitation, either or neither are reported in Table 4. The most commonly reported pain behaviours were slow cautious movements (*N* = 85, 85.9%), splinting (*N* = 76, 76%) and guarding (*N* = 75, 75.6%). The most common agitation behaviours were trying to climb out of bed (*N* = 73, 73.6%), pulling endotracheal (ET) tube (*N* = 36, 36.4%) and seeking attention through movement (*N* = 35, 35.4%).

**TABLE 4:** Reported pain and agitation behaviours.

Item	Pain *N* (%)	Agitation *N* (%)	Either *N* (%)	Neither *N* (%)
Slow cautious movements (*n* = 99)	85 (85.9)	0	7 (7.1)	7 (7.1)
Splinting (*n* = 100)	76 (76)	1 (1)	19 (19)	4 (4)
Guarding (*n* = 99)	75 (75.8)	3 (3)	20 (20.2)	1 (1)
Repetitive touching of area of body (*n* = 99)	55 (55.6)	2 (2)	37 (37.4)	5 (5.1)
Grimacing (*n* = 100)	41 (41)	0	59 (59)	0
Withdrawing (*n* = 98)	36 (36.7)	9 (9.2)	51 (52)	2 (2)
Retraction of upper limbs (*n* = 99)	33 (33.3)	3 (3)	57 (57.6)	6 (6.1)
Arching (*n* = 99)	28 (28.3)	3 (3)	68 (68.7)	0
Diaphoresis (*n* = 97)	27 (27.8)	1 (1)	69 (71.1)	0
Dilated pupils (*n* = 99)	26 (26.3)	5 (5.1)	65 (65.7)	3 (3)
Brow lowering/frowning (*n* = 100)	25 (25)	5 (5)	69 (69)	1 (1)
Rigidity (*n* = 99)	23 (23.2)	3 (3)	73 (73.7)	0
Clenching fists/teeth (*n* = 100)	20 (20)	2 (2)	78 (78)	0
Vocalisation (*n* = 99)	11 (11.1)	3 (3)	84 (84.8)	1 (1)
Inconsolable (*n* = 98)	8 (8.2)	1 (1)	89 (90.8)	0
Desaturation (*n* = 98)	4 (4.1)	11 (11.2)	83 (84.7)	0
Thrashing limbs (*n* = 99)	3 (3)	23 (23.2)	73 (73.7)	0
Seeking attention through movements (*n* = 99)	2 (2)	35 (35.4)	56 (56.6)	6 (6.1)
Fighting ventilator/activation of alarms (*n* = 100)	0	15 (15)	85 (85)	0
Trying to climb out of bed (*n* = 99)	0	73 (73.7)	24 (24.2)	2 (2)
Pulling ET tube (*n* = 99)	0	36 (36.4)	63 (63.6)	0
Irritability (*n* = 99)	0	11 (11.1)	88 (88.9)	0

### 3.3 Facilitators and Barriers

PICU nurses reported that having a designated area for charting pain management (*N* = 90, 93.8%, reporting often or routinely), standardised assessment tools (*N* = 87, 90.6%, reporting often or routinely) and protocols and guidelines (*N* = 83, 86.5%, reporting often or routinely) were the most commonly encountered facilitators of pain management in their units (Table 5). Barriers to pain management were reported less often by the PICU nurses, with the majority reporting barriers occurred never to sometimes for all items. The most commonly experienced barriers to pain management were patient instability (*N* = 22, 22.4% reporting often and routinely), insufficient analgesia dosage prescribed (*N* = 16, 16.5% reporting often or routinely), patient inability to communicate (*N* = 15, 15.3% reporting often and routinely), increased workload (*N* = 14, 14.3% reporting often and routinely) and sedation interfering with pain assessment (*N* = 14, 14.3% reporting often and routinely).

**TABLE 5:** Frequency of facilitators and barriers related to pain management.

Item	Never *N* (%)	Seldom *N* (%)	Sometimes *N* (%)	Often *N* (%)	Routinely *N* (%)
Facilitators					
Hospital pain service consults in the ICU (if available) (*n* = 95)	10 (10.5)	8 (8.4)	25 (26.3)	25 (26.3)	27 (28.4)
Advanced practice nurses are employed by the ICU (*n* = 95)	12 (12.6)	10 (10.5)	15 (15.8)	17 (17.9)	41 (43.2)
Ongoing education on pain is provided (*n* = 95)	1 (1.1)	8 (8.4)	20 (21.1)	37 (38.9)	29 (30.5)
Physicians prescribe adequate doses of analgesia (*n* = 96)	0	5 (5.2)	22 (22.9)	38 (39.6)	31 (32.3)
Appropriate nursing workload (*n* = 96)	0	6 (6.3)	13 (13.5)	48 (50)	29 (30.2)
Enthusiastic and motivated staff (*n* = 96)	0	5 (5.2)	13 (13.5)	50 (52.1)	28 (29.2)
Good communication of pain and analgesic management priorities within ICU team (*n* = 95)	0	3 (3.2)	13 (13.7)	46 (48.4)	33 (34.7)
Protocols and guidelines are in use (*n* = 96)	0	4 (4.2)	9 (9.4)	41 (42.7)	42 (43.8)
Standardised assessment tools are in use (*n* = 96)	0	3 (3.1)	6 (6.3)	39 (40.6)	48 (50)
Pain assessment and management is a unit priority (*n* = 96)	0	2 (2.1)	6 (6.3)	45 (46.9)	43 (44.8)
Designated area for charting pain management (*n* = 96)	2 (2.1)	0	4 (4.2)	31 (32.3)	59 (61.5)
Barriers					
No designated area for charting pain management (Poor electronic health record documentation format) (*n* = 98)	83 (84.7)	9 (9.2)	5 (5.1)	0	1 (1)
Lack of education/familiarity with assessment tools (*n* = 98)	44 (44.9)	33 (33.7)	19 (19.4)	2 (2)	0
Lack of availability of pain assessment tools (*n* = 97)	41 (42.3)	30 (30.9)	24 (24.7)	2 (2.1)	0
Low priority of pain management by ICU team (*n* = 98)	43 (43.9)	39 (39.8)	13 (13.3)	3 (3.1)	0
Lack of protocols/guidelines for pain assessment (*n* = 97)	45 (46.4)	35 (36.1)	12 (12.4)	4 (4.1)	1 (1)
Lack of protocols/guidelines for pain management (*n* = 98)	42 (42.9)	37 (37.8)	13 (13.3)	4 (4.1)	2 (2)
Poor communication of pain and analgesic management priorities within ICU team (*n* = 98)	23 (23.5)	43 (43.9)	24 (24.5)	7 (7.1)	1 (1)
Increased nursing workload (*n* = 98)	10 (10.2)	31 (31.6)	43 (43.9)	13 (13.3)	1 (1)
Sedation interfering with pain assessment (n‐98)	7 (7.1)	33 (33.7)	44 (44.9)	13 (13.3)	1 (1)
Patient inability to communicate (*n* = 98)	8 (8.2)	20 (20.4)	55 (56.1)	15 (15.3)	0
Insufficient analgesia dosage prescribed (*n* = 97)	6 (6.2)	27 (27.8)	48 (49.5)	13 (13.4)	3 (3.1)
Patient instability (*n* = 98)	6 (6.1)	15 (15.3)	55 (56.1)	21 (21.4)	1 (1)

## 4 Discussion

This secondary analysis of a cross‐sectional, descriptive study of pain management in the PICU identifies current pain assessment and management practices as well as barriers and facilitators as reported by PICU nurses. It was found that nurses use pain assessment scales commonly, with more PICU nurses routinely using behavioural pain assessment scales compared to self‐report scales and all PICU nurses valuing physiological variables. When it comes to managing pain, premedication prior to painful procedures was variable, with the insertion of an invasive line being the most common reason for premedicating for pain. To add to this, there were only four behaviours that nurses believed to be more indicative of pain, highlighting the complexity in managing pain and sedation for these children. Lastly, facilitators were reported to be present more commonly than barriers to pain management in the PICU.

Overall, all nurses reported use of pain assessment scales, with a greater emphasis on more routinely using behavioural pain assessment scales versus self‐report scales, which is consistent with the literature for the paediatric population [15, 33, 34]. Additionally, only 67.3% of PICU nurses in this study report attempting to obtain a self‐report of a pain assessment for children who are mechanically ventilated. While many children in the PICU cannot self‐report their pain, LaFond et al. [1] 24‐h observational study found that over half of the children were able to communicate with either full sentences, simple ideas, sounds, gestures, or facial expressions. This points to a need to ensure that PICU nurses are not overly reliant on behavioural cues or physiological variables, which are known to not be reliable but known to be valued when assessing pain [30, 35]. Following Herr et al. [36]'s hierarchy of pain assessment, it is the nurse's ethical duty to first be aware of potential sources of pain and attempt patient self‐report, even using simpler methods, such as nodding, hand gestures, or eye blinks that those who are mechanically ventilated may be able to do.

In the PICU, Baarslag et al. [37] found that the median number of painful procedures experienced per patient per day was 11. In this current study and in the literature, PICU nurses' preprocedural analgesia administration practices are varied [37]. For example, the most common painful procedure in the PICU is reported to be endotracheal suctioning [1, 37], which 87.8% of PICU nurses in this study reported they ‘never to sometimes’ premedicate with analgesia. Endotracheal suctioning is reported to be painful in the PICU by both self‐report and behavioural pain scales [38]. In the adult literature, the experience of endotracheal suctioning is mixed, with some patients reporting it as painful and others not remembering their experience [39, 40, 41]. While those who receive more painful procedures are those that are mechanically ventilated and typically receive continuous intravenous analgesia [1, 37], further research is needed to understand how pain could breakthrough baseline management when a painful procedure is conducted and how best to manage the pain. This also emphasises the importance of appropriate analgosedation management for painful procedures, especially those that occur frequently in the PICU. While there are recommendations for procedural pain management in neonates, there are little to none specifically for critically ill children. Further research is needed to develop evidence‐based practices to provide multimodal pain management prior to procedures in the PICU.

Related to analgosedation, it is known that there is a complex intersection of behaviours that can be indicative of pain or agitation [20]. In this current study, splinting and guarding were shown to be behaviours indicative of pain which is similar to what is found in the adult literature [27, 42]. van Dijk and Ista [16] conducted a Delphi study of PICU experts to develop consensus around behaviours depicting pain, undersedation, iatrogenic withdrawal syndrome and delirium. They reported behaviours, such as facial movements (frowning and quivering chin), vocal sounds (high‐pitched crying and screaming), grasping a specific area of the body, difficult to console, tachypnoea and sweating as more specific to pain. Similarly, in this current study, repetitive touching to an area on the body was found to be more indicative of pain. In this study, over half of the PICU nurses reported that a behaviour could be related to either pain or agitation in 17 out of 22 behaviours listed, which is similar to other studies that reported that most behaviours do overlap [16, 20]. With the large overlap, there is a need to ensure that PICU nurses are aware of the behaviours specific to pain but, perhaps more importantly, ensure that nurses think about pain management first when they assess behaviours that do overlap. Pain management recommendations state if unsure, conduct an analgesic trial first [36]. It is unknown when nurses do encounter overlapping behaviours if they administer analgesia prior to sedative. This along with the lower emphasis on premedication prior to painful procedures found in this study indicates a need to support clinical decision making in real time for nurses at the bedside.

In this current study, nurses perceived having a designated area to chart pain, standardised assessment tools and protocols or guidelines as facilitators in pain management, which aligns with findings in previous studies [18, 43]. This demonstrates the importance of having guidelines in place to help nurses manage these complex patients. In addition, these results indicate that PICU nurses value having clear, allocated charting areas in the electronic health record (EHR) which, when developed well, have been known to improve clinical decision making. However, when the EHR is designed poorly, it can also hinder decision making [44]. This perception on the benefits of a charting system within the EHR presents an opportunity to improve pain management practices through EHRs with integrated pain management guidelines and clinical decision support tools. However, challenges have been reported in making EHR changes in the real world due to differences in each organisations' infrastructure and policies [45, 46]. This would need to be addressed in order to effectively provide clinical decision support tools while balancing the need for standard documentation practices.

Interestingly, as there are barriers reported in the literature [28], PICU nurses here reported experiencing few barriers to pain management either often or routinely in their practice. Of those most reported, patient instability, patient's inability to communicate and sedation interfering with pain assessment are natural, innate features of working in the PICU, and therefore, are difficult to mitigate. However, insufficient analgesia dosing and increased nursing workload were also common barriers reported and could be acted upon in practice. Additionally, having ongoing education was not thought of as a strong facilitator and nurses did not report a lack of education as a barrier. This may indicate a need to move beyond education to implement evidence‐based pain management practices [47, 48]. Implementation strategies should then focus on more active strategies to get evidence‐based pain management practices being done at the bedside, such as decision algorithms and clinician reminders that fit the local context and use local resources [49].

### 4.1 Limitations

This study is not without its limitations. With the use of a self‐report survey, data may not always reflect what is happening in clinical practice. Additionally, survey fatigue may have played a part for those that did not complete the survey. However, this study does further our understanding of the perspectives of pain management for the vulnerable population in paediatric critical care units throughout the United States.

## 5 Implications for Practice

By beginning to understand current context for pain management in the PICU, frontline nurses, nurse educators, and nurse leaders can identify areas for improvement. For instance, there is a need to explore and then implement practice changes related to increasing the use of self‐report pain assessment methods, even if for a child who is mechanically ventilated. Additionally, there is an opportunity to implement practice changes related to analgesic premedication prior to painful procedures. Not only are areas of opportunity identified, but these study results can also help design implementation strategies that combat barriers and enable facilitators, for example, creating changes in the charting system within the EHR to better help the frontline nurse assess and manage pain.

## 6 Conclusion

While there have been other studies related to pain management practices and facilitators and barriers, most have been with either the adult or neonatal populations. This study aimed to describe the unique experience of managing pain in the PICU. We found that PICU nurses are using pain assessment tools to assess pain, with perhaps an overemphasis on behavioural pain assessment tools. There is a need to ensure that nurses are asking for a self‐report of pain even if a child cannot use a formal self‐report pain assessment tool. Future research should look at what are the most appropriate methods to manage pain, especially for commonly occurring procedures. Additionally, with the large amount of overlap between behaviours indicative of pain or agitation reported, clinician decision support tools can be helpful to aid the bedside nurse to navigate evidence‐based probable causes of pain or distress to ensure proper management. Moreover, a primary facilitator reported in this study was the use of the EHR to provide a designated charting area. This could provide an opportunity to further integrate pain management protocols, with clinician decision support into the EHR to help decrease the research to practice gap. Beginning to understand what is happening in practice and what the gaps are is the first step to help better implement and sustain evidence‐based practices.

## Funding

This study was supported by Research Grant from the American Society of Pain Management Nursing.

## Ethics Statement

University of Iowa HawkIRB, #202006492, 6 November 2020.

## Consent

Exempt type was declared by the IRB and informed consent was implied by nurses willing to complete the survey. An information sheet was provided prior.

## Conflicts of Interest

The authors declare no conflicts of interest.

## Supporting information


**Data S1:** Supporting Information.

## Data Availability

Data can be accessed through contact with the corresponding author.

## References

[1] C. LaFond , K. S. Hanrahan , N. L. Pierce , Y. Perkhounkova , E. Laures , and A. McCarthy , “Pain in the Pediatric Intensive Care Unit: How and What Are We Doing?,” American Journal of Critical Care 28, no. 4 (2019): 265–273, 10.4037/ajcc2019836. 31263009

[2] S. Agarwal , D. Classen , G. Larsen , et al., “Prevalence of Adverse Events in Pediatric Intensive Care Units in the United States,” Pediatric Critical Care Medicine 11, no. 5 (2010): 568–578, 10.1097/PCC.0b013e3181d8e405. 20308932

[3] C. B. Groenewald , J. A. Rabbitts , D. R. Schroeder , and T. E. Harrison , “Prevalence of Moderate‐Severe Pain in Hospitalized Children,” Paediatric Anaesthesia 22, no. 7 (2012): 661–668, 10.1111/j.1460-9592.2012.03807.x. 22332912

[4] S. M. Gerik , “Pain Management in Children: Developmental Considerations and Mind‐Body Therapies,” Southern Medical Journal 98, no. 3 (2005): 295–302, 10.1097/01.smj.0000154772.49481.53. 15813156

[5] D. L. Olmstead , S. D. Scott , and W. J. Austin , “Unresolved Pain in Children: A Relational Ethics Perspective,” Nursing Ethics 17, no. 6 (2010): 695–704, 10.1177/0969733010378932. 21097968

[6] A. Twycross , S. Dowden , and J. Stinson , Managing Pain in Children: A Clinical Guide for Nurses and Healthcare Professionals (John Wiley & Sons, 2013).

[7] A. Ekim , “The Post‐Intensive Care Syndrome in Children,” Comprehensive Child and Adolescent Nursing 43, no. 1 (2020): 15–21, 10.1080/24694193.2018.1520323. 30252559

[8] M. E. Hartman , C. N. Williams , T. A. Hall , C. C. Bosworth , and J. A. Piantino , “Post‐Intensive‐Care Syndrome for the Pediatric Neurologist,” Pediatric Neurology 108 (2020): 47–53, 10.1016/j.pediatrneurol.2020.02.003. 32299742 PMC7306429

[9] M. S. M. Ko , P. F. Poh , K. Y. C. Heng , et al., “Assessment of Long‐Term Psychological Outcomes After Pediatric Intensive Care Unit Admission: A Systematic Review and Meta‐Analysis,” JAMA Pediatrics 176, no. 3 (2022): e215767, 10.1001/jamapediatrics.2021.5767. 35040918 PMC8767488

[10] J. C. Manning , N. P. Pinto , J. E. Rennick , G. Colville , and M. A. Q. Curley , “Conceptualizing Post Intensive Care Syndrome in Children‐The PICS‐p Framework,” Pediatric Critical Care Medicine 19, no. 4 (2018): 298–300, 10.1097/pcc.0000000000001476. 29406379

[11] P. Davis , C. Stutchfield , T. A. Evans , and E. Draper , “Increasing Admissions to Paediatric Intensive Care Units in England and Wales: More Than Just Rising a Birth Rate,” Archives of Disease in Childhood 103, no. 4 (2018): 341–345, 10.1136/archdischild-2017-313915. 29084723

[12] American Nurses Association , “ANA Position Statement: The Ethical Responsibility to Manage Pain and the Suffering It Causes,” 2018, https://www.nursingworld.org/globalassets/docs/ana/ethics/theethicalresponsibilitytomanagepainandthesufferingitcauses2018.pdf.

[13] M. Daverio , F. von Borell , A. S. Ramelet , et al., “Pain and Sedation Management and Monitoring in Pediatric Intensive Care Units Across Europe: An ESPNIC Survey,” Critical Care 26, no. 1 (2022): 88, 10.1186/s13054-022-03957-7. 35361254 PMC8969245

[14] A. Ismail , “The Challenges of Providing Effective Pain Management for Children in the Pediatric Intensive Care Unit,” Pain Management Nursing 17, no. 6 (2016): 372–383, 10.1016/j.pmn.2016.08.005. 27756590

[15] E. Laures , C. LaFond , K. Hanrahan , N. Pierce , H. Min , and A. McCarthy , “Pain Assessment Practices in the Pediatric Intensive Care Unit,” Journal of Pediatric Nursing 48 (2019): 55–62. 31325800 10.1016/j.pedn.2019.07.005

[16] M. van Dijk and E. Ista , “Four‐In‐One: A Comprehensive Checklist for the Assessment of Pain, Undersedation, Iatrogenic Withdrawal and Delirium in the PICU: A Delphi Study,” Frontiers in Pediatrics 10 (2022): 887689, 10.3389/fped.2022.887689. 35769214 PMC9234388

[17] R. M. Lebet , N. R. Hasbani , M. T. Sisko , et al., “Nurses' Perceptions of Workload Burden in Pediatric Critical Care,” American Journal of Critical Care 30, no. 1 (2021): 27–35, 10.4037/ajcc2021725. 33385203

[18] M. Rababa , S. Al‐Sabbah , and A. A. Hayajneh , “Nurses' Perceived Barriers to and Facilitators of Pain Assessment and Management in Critical Care Patients: A Systematic Review,” Journal of Pain Research 14 (2021): 3475–3491, 10.2147/jpr.S332423. 34764688 PMC8577531

[19] P. Youngcharoen and S. Aree‐Ue , “A Cross‐Sectional Study of Factors Associated With Nurses' Postoperative Pain Management Practices for Older Patients,” Nursing Open 10, no. 1 (2023): 90–98, 10.1002/nop2.1281. 35762683 PMC9748055

[20] J. Harris , A. S. Ramelet , M. van Dijk , et al., “Clinical Recommendations for Pain, Sedation, Withdrawal and Delirium Assessment in Critically Ill Infants and Children: An ESPNIC Position Statement for Healthcare Professionals,” Intensive Care Medicine 42, no. 6 (2016): 972–986, 10.1007/s00134-016-4344-1. 27084344 PMC4846705

[21] K. Liu , K. Nakamura , H. Katsukawa , et al., “ABCDEF Bundle and Supportive ICU Practices for Patients With Coronavirus Disease 2019 Infection: An International Point Prevalence Study,” Critical Care Explorations 3, no. 3 (2021): e0353, 10.1097/cce.0000000000000353. 33786432 PMC7994035

[22] I. MacDonald , V. de Goumoëns , M. Marston , et al., “Effectiveness, Quality and Implementation of Pain, Sedation, Delirium, and Iatrogenic Withdrawal Syndrome Algorithms in Pediatric Intensive Care: A Systematic Review and Meta‐Analysis,” Frontiers in Pediatrics 11 (2023): 1204622, 10.3389/fped.2023.1204622. 37397149 PMC10313131

[23] Y. Yang , A. Geva , K. Madden , and N. M. Mehta , “Implementation Science in Pediatric Critical Care ‐ Sedation and Analgesia Practices as a Case Study,” Frontiers in Pediatrics 10 (2022): 864029, 10.3389/fped.2022.864029. 35859943 PMC9289107

[24] M. E. Fernandez , G. A. Ten Hoor , S. van Lieshout , et al., “Implementation Mapping: Using Intervention Mapping to Develop Implementation Strategies,” Frontiers in Public Health 7 (2019): 158, 10.3389/fpubh.2019.00158. 31275915 PMC6592155

[25] T. J. Waltz , B. J. Powell , M. M. Matthieu , et al., “Use of Concept Mapping to Characterize Relationships Among Implementation Strategies and Assess Their Feasibility and Importance: Results From the Expert Recommendations for Implementing Change (ERIC) Study,” Implementation Science 10 (2015): 109, 10.1186/s13012-015-0295-0. 26249843 PMC4527340

[26] O. Mala , E. M. Forster , and V. J. Kain , “Neonatal Nurse and Midwife Competence Regarding Pain Management in Neonates: A Systematic Review,” Advances in Neonatal Care 22, no. 2 (2022): E34–e42, 10.1097/anc.0000000000000911. 34224481

[27] L. Rose , L. Haslam , C. Dale , et al., “Survey of Assessment and Management of Pain for Critically Ill Adults,” Intensive & Critical Care Nursing 27, no. 3 (2011): 121–128, 10.1016/j.iccn.2011.02.001. 21398127

[28] K. Alotaibi , I. Higgins , J. Day , and S. Chan , “Paediatric Pain Management: Knowledge, Attitudes, Barriers and Facilitators Among Nurses ‐ Integrative Review,” International Nursing Review 65, no. 4 (2018): 524–533, 10.1111/inr.12465. 29956310

[29] E. L. Laures , J. Williams , and A. McCarthy , “Pain Assessment & Management Decision‐Making in Pediatric Critical Care,” Journal of Pediatric Nursing 73 (2023): e494–e502. 37884405 10.1016/j.pedn.2023.10.020

[30] E. Laures , C. LaFond , B. S. Marie , and A. McCarthy , “Pain Assessment and Management for a Chemically Paralyzed Child Receiving Mechanical Ventilation,” American Journal of Critical Care 32, no. 5 (2023): 346–354. 37652886 10.4037/ajcc2023403

[31] R. C. White‐Traut , “National Pediatric Nurse Scientist Collaborative,” Journal of Pediatric Nursing 34 (2017): 4, 10.1016/j.pedn.2016.12.011. 28159453

[32] P. A. Harris , R. Taylor , R. Thielke , J. Payne , N. Gonzalez , and J. G. Conde , “Research Electronic Data Capture (REDCap)—A Metadata‐Driven Methodology and Workflow Process for Providing Translational Research Informatics Support,” Journal of Biomedical Informatics 42, no. 2 (2009): 377–381, 10.1016/j.jbi.2008.08.010. 18929686 PMC2700030

[33] J. A. Carvalho , D. M. Souza , F. Domingues , E. Amatuzzi , M. C. M. Pinto , and L. M. Rossato , “Pain Management in Hospitalized Children: A Cross‐Sectional Study,” Revista da Escola de Enfermagem da USP 56 (2022): e20220008, 10.1590/1980-220X-REEUSP-2022-0008en. PMC10111388 35652630

[34] J. Haupt , N. Shah , M. Fifolt , et al., “Pain Assessment in Pediatric Emergency Departments: A National Survey,” Pediatric Emergency Care 37, no. 12 (2021): e1145–e1149, 10.1097/pec.0000000000001930. 31815896

[35] S. Shahiri and C. Gélinas , “The Validity of Vital Signs for Pain Assessment in Critically Ill Adults: A Narrative Review,” Pain Management Nursing 24, no. 3 (2023): 318–328. 36781330 10.1016/j.pmn.2023.01.004

[36] K. Herr , A. R. Anderson , C. Arbour , et al., “Pain Assessment in the Patient Unable to Self‐ Report: Clinical Practice Recommendations in Support of the ASPMN 2024 Position Statement,” Pain Management Nursing 25, no. 6 (2024): 551–568, 10.1016/j.pmn.2024.09.010. 39516139

[37] M. A. Baarslag , S. Jhingoer , E. Ista , K. Allegaert , D. Tibboel , and M. van Dijk , “How Often Do We Perform Painful and Stressful Procedures in the Paediatric Intensive Care Unit? A Prospective Observational Study,” Australian Critical Care 32, no. 1 (2019): 4–10, 10.1016/j.aucc.2018.04.003. 29779912

[38] S. D. Duzkaya and S. Kuguoglu , “1251 Assessment of Pain During Endotracheal Suctioning in Pediatric Intensive Care Unit (PICU),” Pediatric Research 68, no. 1 (2010): 620.

[39] P. Gessler , “Pain Relief in Ventilated Preterms During Endotracheal Suctioning. A Randomized Controlled Trial,” Swiss Medical Weekly 138, no. 4344 (2008): 635–645. 19005869 10.4414/smw.2008.12288

[40] E. Gilder , A. Jull , J. Slark , and R. L. Parke , “Patient's Experiences of Endotracheal Tubes and Suction Following Cardiac Surgery,” Nursing in Critical Care 27, no. 2 (2022): 187–194, 10.1111/nicc.12604. 33586305

[41] R. Zeadnih , I. Aljarrah , A. M. Al‐Qaaneh , and M. Atout , “Exploring the Experience of Patients Who Received Mechanical Ventilation Support During Their Intensive Care Unit Stay,” Healthcare (Basel) 12, no. 14 (2024): 1418, 10.3390/healthcare12141418. 39057561 PMC11275606

[42] L. Rose , O. Smith , C. Gélinas , et al., “Critical Care Nurses' Pain Assessment and Management Practices: A Survey in Canada,” American Journal of Critical Care 21, no. 4 (2012): 251–259, 10.4037/ajcc2012611. 22751367

[43] A. Twycross , “Nurses' Views About the Barriers and Facilitators to Effective Management of Pediatric Pain,” Pain Management Nursing 14, no. 4 (2013): e164–e172, 10.1016/j.pmn.2011.10.007. 24315269

[44] O. O. Madandola , R. I. Bjarnadottir , Y. Yao , et al., “The Relationship Between Electronic Health Records User Interface Features and Data Quality of Patient Clinical Information: An Integrative Review,” Journal of the American Medical Informatics Association 31, no. 1 (2023): 240–255, 10.1093/jamia/ocad188. 37740937 PMC10746323

[45] A. Hanson , S. Jackson , and E. Laures , “Implementing an Evidence‐Based Functional Pain Assessment Scale in an Adult Inpatient Unit,” Pain Management Nursing 25, no. 4 (2024): 330–337. 38616456 10.1016/j.pmn.2024.03.004

[46] S. Larson , E. Laures , M. Seo , M. Cox , and M. Wagner , “Evidence‐Based Pain Assessment in Nonverbal Pallliative Care Patients,” Pain Management Nursing 25, no. 2 (2024): 152–159. 38246815 10.1016/j.pmn.2023.12.005

[47] L. E. Ashcraft , D. E. Goodrich , J. Hero , et al., “A Systematic Review of Experimentally Tested Implementation Strategies Across Health and Human Service Settings: Evidence From 2010‐2022,” Implementation Science 19, no. 1 (2024): 43, 10.1186/s13012-024-01369-5. 38915102 PMC11194895

[48] L. Cullen , E. Laures , K. Hanrahan , and S. Edmonds , “The Coat Hook Analogy and the Precision Implementation Approach Solution,” Journal of Perianesthesia Nursing 37, no. 5 (2022): 732–736. 36182248 10.1016/j.jopan.2022.07.009

[49] B. J. Powell , R. S. Beidas , C. C. Lewis , et al., “Methods to Improve the Selection and Tailoring of Implementation Strategies,” Journal of Behavioral Health Services & Research 44, no. 2 (2017): 177–194, 10.1007/s11414-015-9475-6. 26289563 PMC4761530

