# Supplementary material for: Pain Management in Paediatric Critical Care: A Cross‐Sectional Study
Source: Nurs Crit Care. 2026 Jan 20;31(1):e70327. doi: 10.1111/nicc.70327 (PMC12818387; doi:10.1111/nicc.70327)
Supplement: Supplementary file 1 — Data S1: Supporting Information. [file NICC-31-0-s001.pdf]

Supplementary Material 1: Pain Assessment and Management of the Critically ill Child Survey

Please take the time to answer the following survey about your general pain assessment and management practices, and the barriers and facilitators to assessing and managing pain in your practice.

The following questions relate to ALL patients seen in the PICU (mechanically ventilated and non-mechanically ventilated).

1. How likely are you to provide pre-emptive analgesia prior to the following procedures?

Procedure	Not at all	Minimally	Somewhat	Moderately	Extremely
Patient repositioning (Side to side)					
ET suctioning					
Wound care					
Drain removal					
Invasive line placement					
Spontaneous breathing trial					
Imaging					

The following questions relate to patients *able to communicate* verbally or via other means (holding up fingers, pointing, etc.).

1. Do you use a pain assessment tool for patients <u>*able to communicate*</u>?
   a. Yes
   b. No
   If yes, please identify which tools (s) you currently use (select all that apply):
      a. Numeric Rating Scale
      b. FACES Pain Scale-Revised
      c. Visual analog Scale
      d. Wong-Baker FACES Scale
      e. Verbal Descriptor Scale
      f. Other______________

   If you do not use a pain assessment tool, please describe your method for assessing pain for patients able to self-report: ____

2. How frequently do you use a pain assessment tool for *patients able to communicate*?
   a. Never
   b. Seldom
   c. Sometimes
   d. Often
   e. Routinely

3. Do you attempt to obtain a self-report of pain for a child that is mechanically ventilated?
   a. Yes
   b. N

The following questions relate to patients *unable to communicate* verbally or via other means (due to age, sedation, pharmacological paralysis, developmental delay).

1. Do you use a pain assessment tool for patients <u>*unable to communicate*</u>?
   a. Yes
   b. No

   If yes, please identify which tool(s) you currently use (select all that apply):
   a. FLACC
   b. COMFORT-B
   c. NIPS
   d. CRIES
   e. BPS
   f. Other_______________

   If you do not use a pain assessment tool, please describe your method of assessing pain for patients *unable to communicate* their pain: ____

2. How frequently do you use a pain assessment tool for patients *unable to communicate* their pain?

f. Never
g. Seldom
h. Sometimes
i. Often
j. Routinely

3. Please rate each of the following behaviors as indicators for pain, agitation (under sedation), either pain or agitation, or neither pain nor agitation.

Behavior	Pain	Agitation	Either	Neither
Grimacing				
Rigidity				
Vocalization				
Brow lowering/frowning				
Fighting ventilator/activation of alarms				
Splinting				
Clenching fists/teeth				
Slow cautious movements				
Retraction of upper limbs				
Trying to climb out of bed				
Repetitive touching of area of body				
Seeking attention through movements				
Pulling ET tube				
Irritability				
Thrashing limbs				
Withdrawing				
Guarding				
Arching				
Inconsolable				
Diaphoresis				
Dilated pupils				
Desaturation				
Other_________________				

4. Please select physiological indicators you feel are indicative of pain (select all that apply)?

a. Heart rate
b. Blood pressure
c. Pupil size
d. Diaphoresis
e. None
f. Other_____________________

<u>The following questions pertain to the barriers and facilitators in your ability to assess and manage pain in your practice.</u>

1. Please score how often the following situations ***impede*** your ability to effectively assess and manage pain.

Item	Never	Seldom	Sometimes	Often	Routinely
Increased nursing workload					
Lack of availability of pain assessment tools					
Lack of education/familiarity with assessment tools					
Patient instability					
Patient inability to communicate					

Lack of protocols/guidelines for pain assessment					
Low priority of pain management by ICU team					
No designated area for charting pain management (Poor documentation format)					
Sedation interfering with pain assessment					
Poor communication of pain and analgesic management priorities within ICU team					
Lack of protocols/guidelines for pain management					
Insufficient analgesia dosage prescribed					
Other_____________					

2. Please identify the frequency with which the following ***enable*** delivery of effective pain practice.

Item	Never	Seldom	Sometimes	Often	Routinely
Appropriate nursing workload					
Pain assessment and management is a unit priority					
Enthusiastic and motivated staff					
Standardized assessment tools are in use					
Protocols and guidelines are in use					
Designated area for charting pain management					
Physicians prescribe adequate doses of analgesia					
Good communication of pain and analgesic management priorities within ICU team					
Ongoing education on pain is provided					
Advanced practice nurses are employed by the ICU					
Hospital pain service consults in the ICU (if available)					
Other ________________					

<u>Demographics</u>

1. What is your age in years? ___________
2. Please specify your ethnicity/race. (Check all that apply).
   a. White
   b. Hispanic/Latinx
   c. Black/ African American
   d. Native American/ American Indian
   e. Asian/ Pacific Islander
   f. Other (please specify) ___________
   g. I wish to not specify
2. What is your gender?
   a. Male
   b. Female
   c. I wish not to specify
   d. Other (please specify)_________
3. How long have you worked in the PICU?
   a. Less than a year
   b. 1 to 2 years
   c. 2 to 5 years
   d. 5 to 10 years

e. Greater than 10 years

4. What is the highest level of education you have received?
    a. Associates Degree in Nursing (RN, AD)
    b. Bachelor's Degree (BSN, BA)
    c. Master's Degree in Nursing (MSN, MA)
    d. Doctoral degree (DNP or PhD)
5. Which of the following best describes your typical work schedule/shifts?
    a. Days (8-hour shifts)
    b. Evenings (8-hour shifts)
    c. Nights (8-hour shifts)
    d. Days (12-hour shifts)
    e. Nights (12-hour shifts)
    f. Weekend package (Day shift)
    g. Weekend package (Night shift)
    h. Combination of different shift types (Rotating)
    i. PRN
    j. Other
6. We are interested in talking with a subgroup of PICU nurses about their pain assessment and management practices for a mechanically ventilated child. This would involve a one-hour interview via Zoom or a phone call. Compensation would include… Would you be willing to participate in an interview?
    a. Yes
    b. No

Thank you for completing the survey and for your time.
